# A Case of Rituximab-Induced Necrotizing Fasciitis and a Review of the Literature

**DOI:** 10.1155/2017/6971027

**Published:** 2017-09-26

**Authors:** Abdullateef Abdulkareem, Ryan S. D'Souza, Oluwaseun Shogbesan, Anthony Donato

**Affiliations:** ^1^Department of Medicine, Reading Hospital, West Reading, PA, USA; ^2^Department of Anesthesiology, Mayo Clinic Hospital, Rochester, MN, USA

## Abstract

Necrotizing fasciitis is a fulminant soft tissue infection characterized by rapid progression and high mortality. Rituximab is a generally well-tolerated immunosuppresive medication used for B-cell malignancies and some rheumatological disorders. We report a case of a 69-year-old male with chronic lymphocytic leukemia who suffered necrotizing fasciitis of his left lower extremity secondary to* Clostridium septicum* 7 weeks after treatment with rituximab. Despite immediate intravenous antimicrobial therapy and emergent fasciotomy with extensive debridement, his hospital course was complicated by septic shock and he required an above-the-knee amputation. Physicians need to be aware of the possibility of necrotizing fasciitis in patients presenting with skin infections after rituximab therapy.

## 1. Introduction

Necrotizing fasciitis (NF) is a rare, fulminant soft tissue infection characterized by rapidly progressive and extensive destruction of the skin, subcutaneous fat, and fascia [1]. While the incidence of NF is very low (0.4 cases per 100,000 adults) [1], it is unfortunately associated with a high mortality rate, ranging between 25 and 73% [1, 2]. Urgent and aggressive surgical debridement is needed, as delayed or inadequate debridement has been linked with increased mortality [2–4]. Rituximab is an anti-CD20 monoclonal antibody that leads to immunosuppression via depletion of CD20-positive B lymphocytes, and is indicated for treatment of B-cell hematological malignancies [5, 6] and rheumatological conditions including systemic lupus erythematosus (SLE) [7] and rheumatoid arthritis (RA) [8]. We present a case of NF that occurred seven weeks after a course of rituximab infusions in a patient with chronic lymphocytic leukemia (CLL), and review similar relevant cases of rituximab-associated NF.

## 2. Case Report

A 69-year-old male with a history of CLL presented to the emergency department with a 2-day history of progressively worsening left lower limb pain, fever, chills, and malaise. He denied any trauma to his legs, skin breaks, bug bites, nausea, vomiting, weight loss, sick contacts, lymphadenopathy, neurological deficits, or recent travel history. There was no documented history of diabetes, tobacco use, alcoholism, or prior severe infections.

The patient was diagnosed with CD-38 negative CLL eight years prior to admission and was treated 2 years after initial diagnosis with 6 cycles of bendamustine and rituximab after which he was in complete remission. Approximately 11 weeks prior to admission, he was retreated with a 4-week course of rituximab for a resurgence of his CLL, characterized by leukocytosis, anemia, thrombocytopenia, and hypogammaglobulinemia (IgG: 379 mg/dL (normal: 767–1590 mg/dL)). This course was completed 7 weeks before admission.

On initial assessment, he was afebrile (36.5°C) and other vital signs were normal. Left lower limb examination revealed moderate tenderness to palpation but no erythema or swelling. However, within 7 hours of presentation, the patient was noted to have a fever of 39.2°C, and pain in his left lower extremity worsened considerably accompanied with extensive tissue induration, new erythema extending from the left ankle to the knees, and subcutaneous emphysema.

Laboratory results were notable for white blood count (WBC) of 5,800 c/*μ*L (4,800–10,800 c/*μ*L), absolute neutrophil count of 800 c/*μ*L, and platelet count of 61,000 c/*μ*L (130,000–400,000 c/*μ*L). Lactic acid was elevated at 3.6 mEq/L (0.5–2.2 mEq/L). C-reactive protein was elevated at 2.95 mg/dL (0.00–0.70 mg/dL). Creatine phosphokinase (CK) level was elevated at 5185 IU/L (30–223 IU/L).

**Figure 1:**
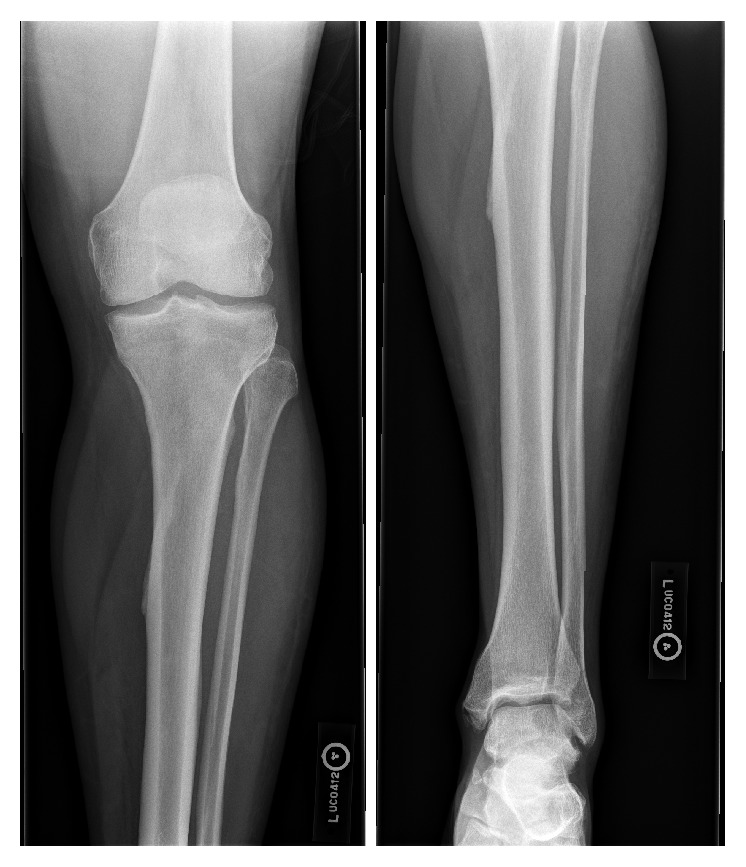
Initial X-ray of the left tibia and fibula is displayed. There is mild tibial plafond spurring. No other acute abnormalities are identified.

**Figure 2:**
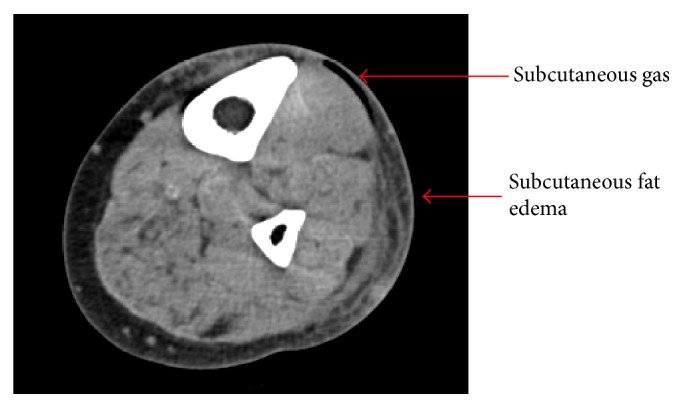
CT scan of the left lower extremity is displayed. Small amount of gas and subcutaneous fat edema present in anterior and lateral leg.

Initial X-rays of the lower extremity (Figure 1) and lower extremity arterial duplex studies were normal. CT scan performed three hours after initial X-rays however showed a small amount of gas and subcutaneous fat edema in anterolateral leg, concerning for NF (Figure 2). Two out of four blood cultures grew* Clostridium septicum* by day 2.

The patient was started on intravenous broad-spectrum antibiotics including vancomycin and piperacillin-tazobactam. He underwent emergent fasciotomy of the left lower extremity with complete excisional debridement of the anterior compartment and counterincisions of the left dorsal foot and left mediolateral thigh. However, he still required a guillotine left below the knee amputation the following day. Pathological analysis of the excised tissue showed necrotic fibrous tissue consistent with fascia and associated necrotic skeletal muscle.

Postoperatively, the patient was transferred to the intensive care unit due to septic shock requiring norepinephrine infusion to maintain blood pressure; this was successfully weaned by the first postoperative day. During the next three weeks, the patient had a total of seven debridement procedures along with an above-the-knee amputation. He stayed in the hospital for a total of three weeks prior to discharge to a rehabilitation facility.

## 3. Discussion

We describe the case of a patient who developed* Clostridium* septicemia manifesting as NF requiring amputation. Our patient was elderly (69 years old) with CLL complicated by hypogammaglobulinemia, which may itself have predisposed him to NF [18]; however the temporal relationship to rituximab therapy suggests that the treatment may have increased his susceptibility [19]. In the eight other patients we identified in the literature, all developed NF within 2 months of their last rituximab treatment (Table 1) which is congruent with prior descriptions of a temporal relationship between rituximab and severe infections in rheumatologic disease registries [19, 20]. However a systematic review and meta-analysis of the risk of infections in patients with lymphoma treated with rituximab did not demonstrate a relative increase in the incidence of severe infections when added to standard chemotherapy [21], and a similar study did not find increased risk of severe infections in RA patients [22].

Besides rituximab-induced necrotizing fasciitis, other drug-related necrotizing fasciitis cases in immunocompromised patients have been previously reported in the literature, including associations with systemic corticosteroids [23], tumor necrosis factor-alpha (TNF-*α*) inhibitors [24], nonsteroidal anti-inflammatory drugs [25], and other immunosuppressants such as tocilizumab [26] and bortezomib [27]. Although it is unclear whether the primary precipitating factor for necrotizing fasciitis is from immunosuppressive pharmacologic therapy, preexisting immunodeficiency, or malignancy, the temporal occurrence of necrotizing fasciitis was noted to occur closer to administration of immunosuppressant therapy in most cases.

NF is classified into two groups: type 1, a polymicrobial infection from anaerobes including* Clostridia*, gram-negative rods, and gram-positive cocci, and type 2, a monomicrobial infection often due to Group A Streptococcus [2]. Risk factors for NF infection include immunosuppression, diabetes mellitus, trauma, or recent surgery and intravenous drug use [18]. Interestingly, although polymicrobial type 1 NF is commonly associated with immune impairment, our patient and all the cases we identified had monomicrobial NF. This suggests that the mechanism may have been initial bacteremia and seeding of that organism in otherwise viable tissue rather than overgrowth of polymicrobial bacteria in tissue with limited blood supply that typically characterizes type 1 NF.

Monomicrobial infection is usually associated with gram-positive infections; however gram-negative monomicrobial NF is being increasingly reported [28]. Sixty-seven percent (67%) of the cases we reviewed had gram-negative organisms implicated. Yahav et al. in a single-center study compared gram-negative and gram-positive monomicrobial NF and found that gram-negative monomicrobial NF was associated with mostly immunocompromised patients and higher mortality rates [29]. Our patient had a monomicrobial infection with* Clostridium septicum*, a gram-positive anaerobe.* Clostridium septicum* NF has been linked with underlying malignancy, particularly colon cancer and hematological malignancies [30]. In a recent review, 61% of adult patients with* Clostridium septicum* NF had gastrointestinal malignancies [30]. Notably, our patient had a normal colonoscopy two years prior to admission.

Of those reporting fatalities in our review, five of the eight patients (63%) died. The death rate in this case series was in the higher range of what is reported in the literature, which may be attributable to the impaired immune response in patients receiving anti-B-cell therapies. Lee et al. found that patients with gram-negative NF presented with more severe sepsis [31].

## 4. Conclusion

Rituximab used in conjunction with steroids and other immunosuppressant agents may increase the risk of serious infections such as NF; other important factors which increase the risk of serious infections while on rituximab include elderly age and hypogammaglobulinemia. Emergent surgical consultation and fasciotomy should be performed, as delay in treatment has been associated with increased mortality. Further studies should investigate the relationship between rituximab and NF.

## Figures and Tables

**Table 1:** Cases of patients developing NF after rituximab therapy.

S/N	Study	Condition	Age/sex	Dosing protocol	Cycle, dose	Onset of NF from last dose	Organism	Location of NF	Concurrent medication	Comorbidities	Outcome
1	Current study	CLL	69/M	375 mg/m^2^ ×4	2, 4	7 weeks	*Clostridium septicum*	LLE	None	None	Survived

2	Lazzeri et al. [9, 10]	CLL	44/M	NA	NA	NA	*Pseudomonas aeruginosa*	Periorbital	Fludarabine Cyclophosphamide	NA	Survived

3	Guerrero et al. [11]; Wong et al. [12]	SLE	21/M	NA	NA	NA	*Acinetobacter baumannii*	Abdomen Bil flanks Thigh Scapula	Steroids	NA	Died

4	Krieger et al. [13]	NHL, follicular	48/F	375 mg/m^2^ (weekly)	1, 4	1 month	*Pseudomonas aeruginosa*	Left lower extremity	NA	NA	Died

5	Krieger et al. [13]	NHL, follicular	59/M	375 mg/m^2^ (weekly) Rituximab stopped after 3 doses.	1, 3	NA	*Escherichia coli (E. coli)*	Scrotum	Etoposide, cisplatin, methyl prednisone, cytosine, and arabinoside	NA	Died

6	Looney et al. [14]	SLE	37/F	375 mg/m^2^ ×4 (weekly)	1, 4	6 weeks	Group A *Streptococcus*	Elbow	Methotrexate Steroids	Septic arthritis	N/A

7	Chen and Isenberg [15]	SLE	26/F	1 g weekly	1, 2	2 months	*Pseudomonas aeroginosa*	LLE	Hydroxychloroquine Azathioprine Cyclophosphamide Steroid	AVN below Digital ischemia *E. coli* UTI	Survived

8	Morita et al. [16]	Waldenstrom's macroglobulinemia	76/M	375 mg/m^2^ (weekly)	1, 1	2 weeks	*Klebsiella pneumonia*	RLE	Bortezomib Dexamethasone	Diarrhea	Died

9	Raghuvanshi and Menon [17]	Rheumatoid arthritis	62/F	NA	1, 2	2 weeks	Group A *Streptococcus*	LUE	Prednisone	NA	Died

AVN = avascular necrosis; Bil = bilateral; CLL = chronic lymphocytic leukemia; F = female; LLE = left lower extremity; LUE = left upper extremity; M = male; NA = not available; NF = necrotizing fasciitis; NHL = non-Hodgkin's lymphoma; RLE = right lower extremity; SLE = systemic lupus erythematosus; S/N = serial number; UTI = urinary tract infection.
